# Single-axon level morphological analysis of corticofugal projection neurons in mouse barrel field

**DOI:** 10.1038/s41598-017-03000-8

**Published:** 2017-06-06

**Authors:** Congdi Guo, Jie Peng, Yalun Zhang, Anan Li, Yuxin Li, Jing Yuan, Xiaofeng Xu, Miao Ren, Hui Gong, Shangbin Chen

**Affiliations:** 1 Britton Chance Center for Biomedical Photonics, Wuhan National Laboratory for Optoelectronics-Huazhong University of Science and Technology, Wuhan, 430074 China; 2 Key Laboratory for Biomedical Photonics of Ministry of Education, Department of Biomedical Engineering, Huazhong University of Science and Technology, Wuhan, 430074 China

## Abstract

Corticofugal projection neurons are key components in connecting the neocortex and the subcortical regions. In the barrel field, these neurons have various projection targets and play crucial roles in the rodent whisker sensorimotor system. However, the projection features of corticofugal projection neurons at the single-axon level are far from comprehensive elucidation. Based on a brain-wide positioning system with high-resolution imaging for Thy1-GFP M-line mice brains, we reconstructed and analyzed more than one hundred corticofugal projection neurons in both layer V and VI of barrel cortex. The dual-color imaging made it possible to locate the neurons’ somata, trace their corresponding dendrites and axons and then distinguish the neurons as L5 type I/II or L6 type. The corticofugal projection pattern showed significant diversity across individual neurons. Usually, the L5 type I neurons have greater multi-region projection potential. The thalamus and the midbrain are the most frequent projection targets among the investigated multidirectional projection neurons, and the hypothalamus is particularly unique in that it only appears in multidirectional projection situations. Statistically, the average branch length of apical dendrites in multi-region projection groups is larger than that of single-region projection groups. This study demonstrated a single-axon-level analysis for barrel corticofugal projection neurons, which could provide a micro-anatomical basis for interpreting whisker sensorimotor circuit function.

## Introduction

The barrel cortex has been recognized as a classical model for studying the neural circuits that connect the neocortex and the subcortical region^1^. As a type of neocortical excitatory neurons, pyramidal neurons in the barrel field are fundamental components of the whisker sensorimotor system^2, 3^. Besides the contralateral cortex projections through the corpus callosum (cc), the long-distance axons of major pyramidal neurons may project to a mass of subcortical regions, such as the striatum, the thalamus, the superior colliculus and the pons^4^. Thus, those pyramidal neurons are defined as corticofugal projection neurons^5^. To interpret brain wiring, it is critical to investigate the corticofugal projections to understand neuroanatomical connectivity^6^.

Recently, great progress has been made in brain circuitry^7–9^. Using anterograde and retrograde tracing, such as histological staining or virus vectors, allows research on corticofugal projections at different brain regions in terms of anatomy^10–12^. In particular, the Allen Mouse Brain Connectivity Atlas (http://connectivity.brain-map.org/)^13^ mapped whole-brain region-to-region connectivity intensities at mesoscale using an adeno-associated virus (AAV) tracer and a serial two-photon (STP) tomography system. As a unique resource for neural circuit investigation, this atlas includes a large amount of information on corticofugal projections. Nevertheless, most work on corticofugal projections considers the entire neuronal population (or a particular cell type, depending on the labeling method) at a certain brain region as an ensemble to obtain statistical projection distribution patterns^12, 14, 15^. Some single-neuron studies^16–18^ that apply sparse labeling techniques have not accumulated sufficient reconstruction resources with regard to the requirements of neurite-level connectomics^19^. As a consequence, the microscopic cortico-subcortical connectivity remains largely unknown. To understand the mechanisms of integration and interaction in circuits, analyzing projections at the single-axon level is essential to discover the arborization diversities and target preferences among the same cell-types. The reconstruction of long-range circuits with axonal resolution is a big challenge that depends heavily on both labeling (appropriate labeling density) and imaging (high radial and axial resolution) techniques^20^.

To resolve the neural circuit that connects the barrel field and the subcortical target at microscopic resolution, we resorted to our newly developed brain-wide positioning system (BPS)^21^. Our previous work revealed neurons with distinct axon projection targets that show some differentiation of dendritic morphology. By taking advantage of the co-localization feature of BPS, we reconstructed and located more than one hundred corticofugal projection neurons in layer V/VI of the barrel field cortex. By defining different projection modes based on axon ramification, we focused on the pyramidal neurons that have two or more projection targets. This work intends to bring a novel and precise perspective for investigating subcortical connectivity of the barrel field.

## Results

### Classification and projection

**Figure 1:**
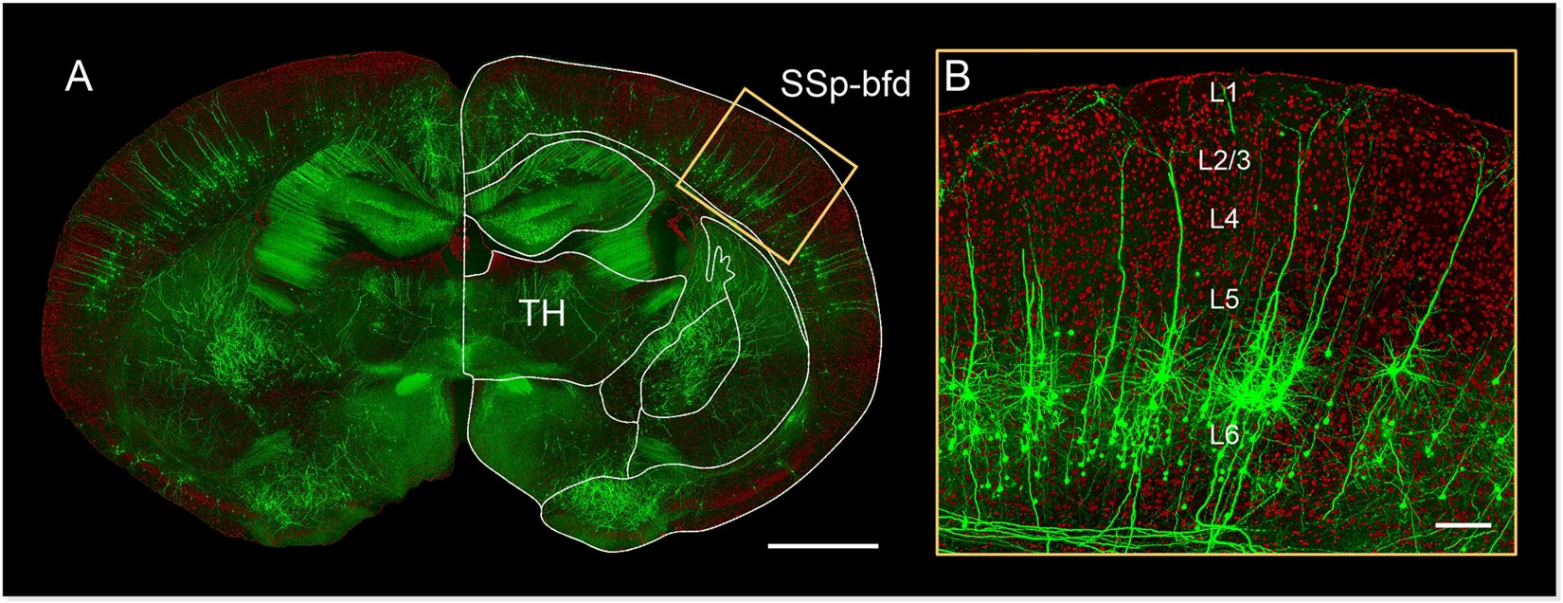
Location of the barrel field and the local morphology of the pyramidal neurons. (**A**) The merged image of the GFP-channel (green, thickness of projection: 300 μm) and the PI-channel (red, thickness of projection: 2 μm). The superimposed outline is used for brain area localization. SSp-bfd indicates barrel-related primary somatosensory cortex. TH represents the thalamus. Scale Bar = 1 mm. (**B**) Enlarged view of the yellow bounding box in (**A**) with rotation, which illustrates the cortical cytoarchitecture and the fine morphology of pyramidal neurons. Scale Bar = 100 μm.

**Figure 2:**
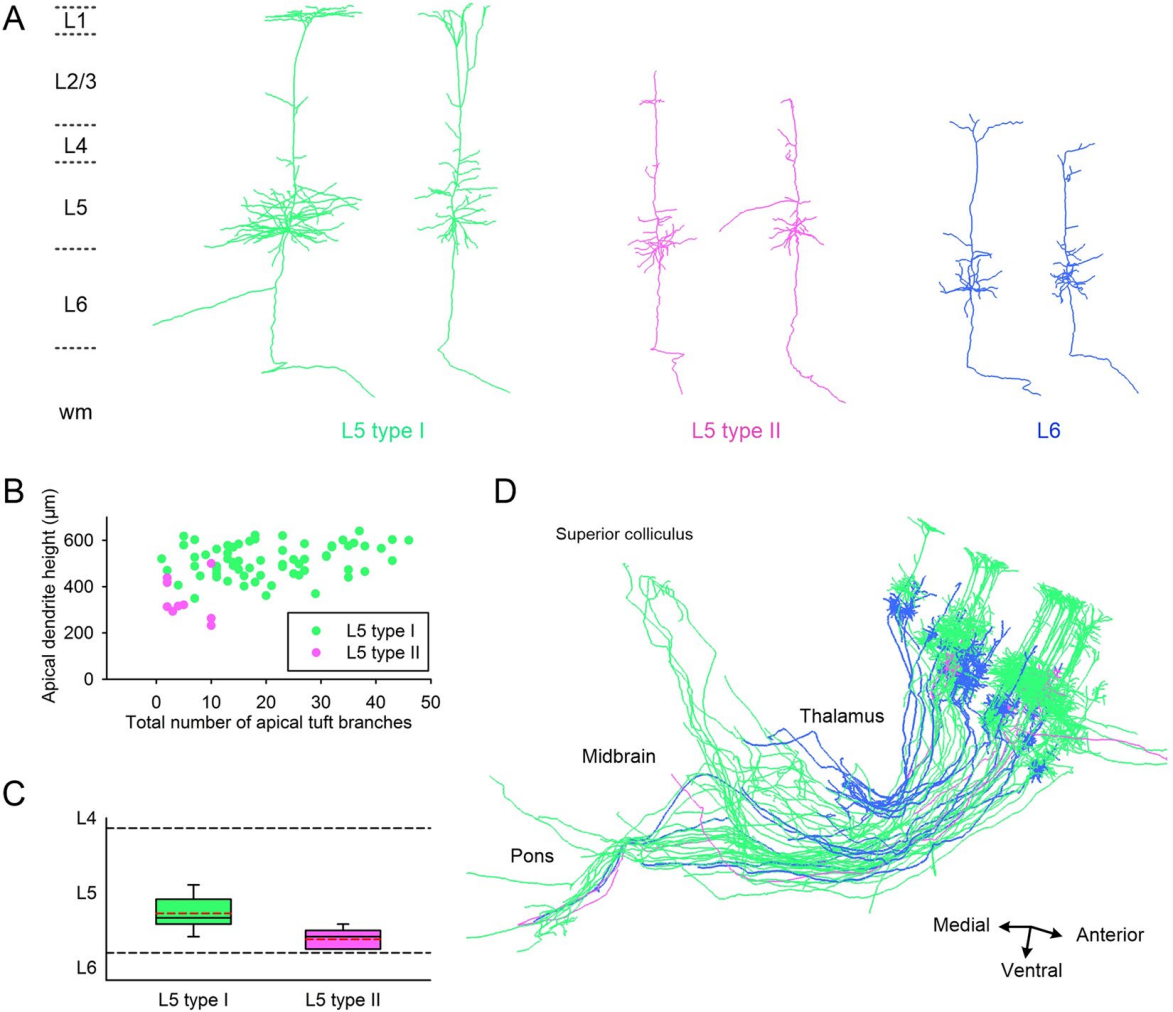
Three typical morphology-based types of pyramidal neurons (**A**) are reconstructed and encoded with different colors. (**B**) Branch number of the apical tuft versus apical dendrite height in L5 type I and II. (**C**) Distribution boxes of relative soma locations of L5 type I and II, where the red dotted line represents the mean value. L5 type I: n = 67; L5 type II: n = 9. (**D**) The general spatial projection of 65 pyramidal neurons from the same hemisphere of one brain dataset. L5 type I: green, n = 39; L5 type II: purple, n = 5; L6: blue, n = 21.

Using the dual-color BPS system, we acquired high resolution datasets at a voxel size of 0.32 × 0.32 × 2 μm with two Thy1-GFP M-line mice (Fig. 1). We reconstructed all 107 corticofugal projection neurons in layers V (n = 76) and VI (n = 31) of the barrel field. We determined the layer where the somata were located from original resolution slices and 50-μm-thick maximal intensity projection images of the propidium iodide (PI) stained channel dataset. Then, for the layer V pyramidal neurons, we classified them into type I and type II according to the morphological features of apical dendrites^22^. In general, the apical dendrites of type I neurons reach layer I, but type II neurons do not (Fig. 2A). This is also reflected in the discrepancy of absolute apical dendrite height (512.8 ± 8.7 μm vs 343.7 ± 29.5 μm, *p*-value = 6.79 × 10^−9^, two-tailed). Moreover, the type I neurons always have a thick apical tuft but the type II neurons only have sparse apical arbors (Fig. 2A). We measured the number of apical tuft branches to verify this criterion. Although the overall difference was significant (20.7 ± 1.4 vs 5.3 ± 1.2, *p*-value = 6.76 × 10^−10^, two-tailed), a considerable overlap remained between the groups (Fig. 2B). Therefore, based on the apical dendrites’ reaching to layer I, we distinguished layer V pyramidal neurons into two sub-populations (Fig. 2B; one outlier of type II occurred because its apical dendrites were extended closely to layer I). The numbers of type I and type II neurons are 67 and 9, respectively. Most somata of L5 neurons were located in L5b (Fig. 2C), and the relative soma positions of the type II neurons were deeper in layer V than were type I neurons (*p*-value = 3.62 × 10^−4^, two-tailed). Considering their relative homogeneity of dendritic morphology, we did not subclassify L6 pyramidal neurons (Fig. 2A).

**Figure 3:**
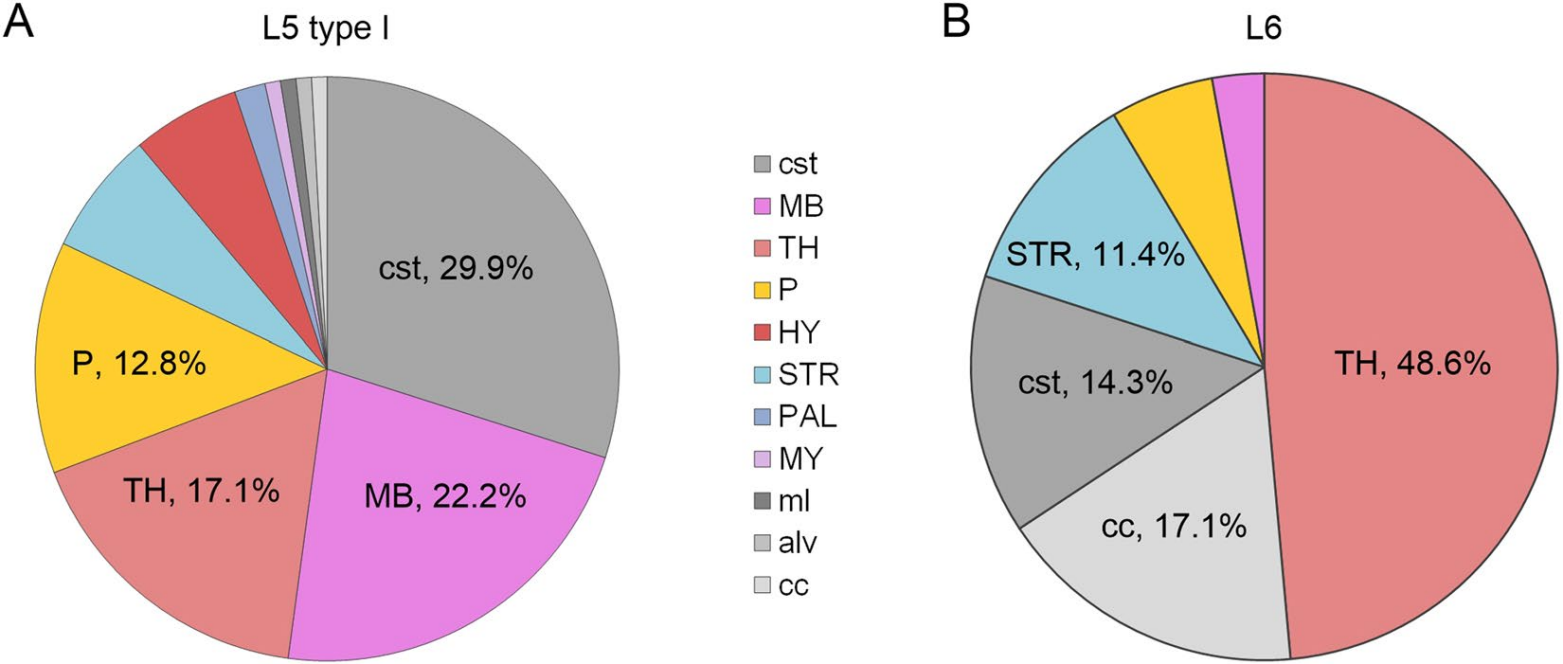
Pie charts on the fractional occurrence frequency of a certain projection target region to all regions observed by the axon tip. Results of L5 type I neurons (**A**) and L6 neurons (**B**). The frequency values ranked in descending order in a clockwise direction. Abbreviations: cst – corticospinal tract; MB – Midbrain; TH –Thalamus; P – Pons; HY – Hypothalamus; STR – Striatum; PAL – Pallidum; MY – Medulla; ml – medial lemniscus; alv – alveus; cc – corpus callosum.

Since all of the 107 corticofugal projection neurons have clear subcortical projections (Fig. 2D illustrates one of the two datasets), we located all of the reconstructed axon distal terminals according to the PI channel datasets (more details in Methods) by referring to the Allen Reference Atlas^23^. The occurrence frequency of a certain projection region for one neuron is 0 or 1. Figure 3 shows the average frequencies of the brain regions where the axon distal terminals of all L5 type I and L6 pyramidal neurons are located. The pie charts show significant differences between the cell types in the distribution of corticofugal projections, including the projection regions and the corresponding proportion values. The corticospinal tract (cst) and the thalamus (TH) are the most frequent projection regions of L5 type I and L6 pyramidal neurons, respectively. In addition, the midbrain (MB) and the thalamus (TH) also show a high occurrence frequency (>17%) in L5 type I pyramidal neurons. Because the sample size of the type II neurons was smaller than 10, we did not list the projection frequencies to the different regions. In fact, most of the L5 type II neurons project to the striatum (STR) or the corticospinal tract (cst) in this study (Supplementary Table S1).

### Analysis of projection modes

With annotation of the corticofugal projections, we found that a considerable amount of neurons have multi-region projections, i.e., at least two targeting regions (Fig. 2D). We distinguished the different “projection modes” of a corticofugal projection neuron according to its projection regions. For example, we used “corticostriatal” or “corticothalamic” for those single-region projection neurons that only project to the striatum or the thalamus, respectively, and we used “TP” for multidirectional projection neurons that project to the thalamus and the pons. We abbreviated several names of the main projection regions: C-cst, corticospinal tract; S-STR, striatum; T-TH, thalamus; H-HY, hypothalamus; M-MB, midbrain; P, pons. The above region abbreviations were sorted by ascending spatial distance of the barrel field to the region. We found that 43% of L5 type I neurons had multi-region corticofugal projections and 19% of L5 type I neurons had more than two regions of corticofugal projection. In contrast, only 10% of L6 neurons had multidirectional corticofugal projections and none of the L6 neurons had more than three directions of corticofugal projections (Supplementary Table S2).

**Figure 4:**
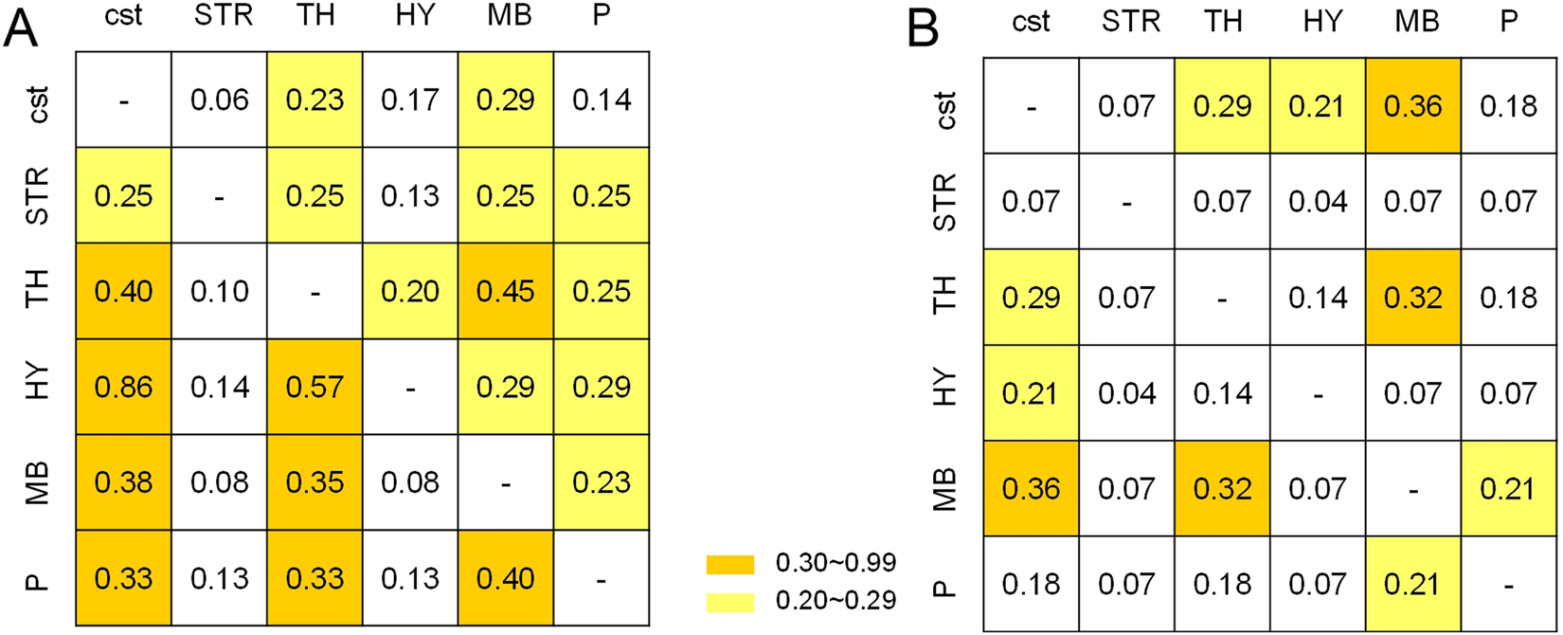
Quantification of the projection mode of L5 type I neurons. (**A**) The collaboration matrix quantifies the “posterior probability” of another projection region given the neuron projection to the current region. Taking the value in the second row and the third column as an example: 0.25 = *Num*_*ST*_/*Num*_*S*_, *Num*_*ST*_ represents the number of multidirectional projection neurons to include the mode “ST”. *Num*_*S*_ represents the number of neurons that project to the striatum. Similarly, the value in the third row and the second column can be calculated by: 0.10 = *Num*_*ST*_/*Num*_*T*_. (**B**) The coexistence matrix measures the occurrence frequency of two directional projection modes. Every element of the matrix represents the ratio of some neurons projecting to the two regions to all the neurons having at least two projection regions (n = 28).

The L5 type I neurons showed different projection profiles under multi-region projection mode. Table 1 lists the ratio values of the multi-region projection neurons that target a certain region to all the neurons that project to the same region. The two region projection data show that the ratio in the striatum is the lowest, which is less than 0.4, but the thalamus, hypothalamus, midbrain and pons all have ratios greater than 0.6. Specifically, the hypothalamus has a 100% two-region projection ratio. This means that if a L5 type I neuron has projections to the hypothalamus, then it must have collateral projections to other regions. Additionally, the multi-region projection (more than two) ratio of the hypothalamus is greater than other regions. We pooled the L5 type I neurons that had no less than two directions of corticofugal projection and measured the two occurrence frequency parameters. A collaboration matrix was used to quantify the “posterior probability” of another projection region given the neuron projection to the current region (Fig. 4A). For example, the neuron projecting to the hypothalamus (HY) has extra projections to the corticospinal tract (cst) with a probability of 0.85. Another coexistence matrix was applied to measure the ratio of some neurons projecting to the two regions to all the neurons having at least two projection regions (Fig. 4B). The results suggested that the cst, TH and MB are key targets of the multidirectional projection mode: “CM” and “TM” modes occurred most frequently, and TH and MB had greater “extra projection” ratios in all regions. The abovementioned “strong synergistic” feature of the hypothalamus was also reflected in the high values of the collaboration matrix.

**Table 1:** Multi-region projection ratio of L5 type I neurons projecting to the target regions.

Projection region	Ratio of multidirectional projection (region #> = 2)	Ratio of multidirectional projection (region #> = 3)
cst	0.51	0.23
STR	0.38	0.38
TH	0.75	0.45
HY	1.00	0.71
MB	0.73	0.31
P	0.67	0.47

The denominator in the ratio only sums up the number of neurons that project to the investigated region.

### Correlation between projection and dendrite complexity

**Figure 5:**
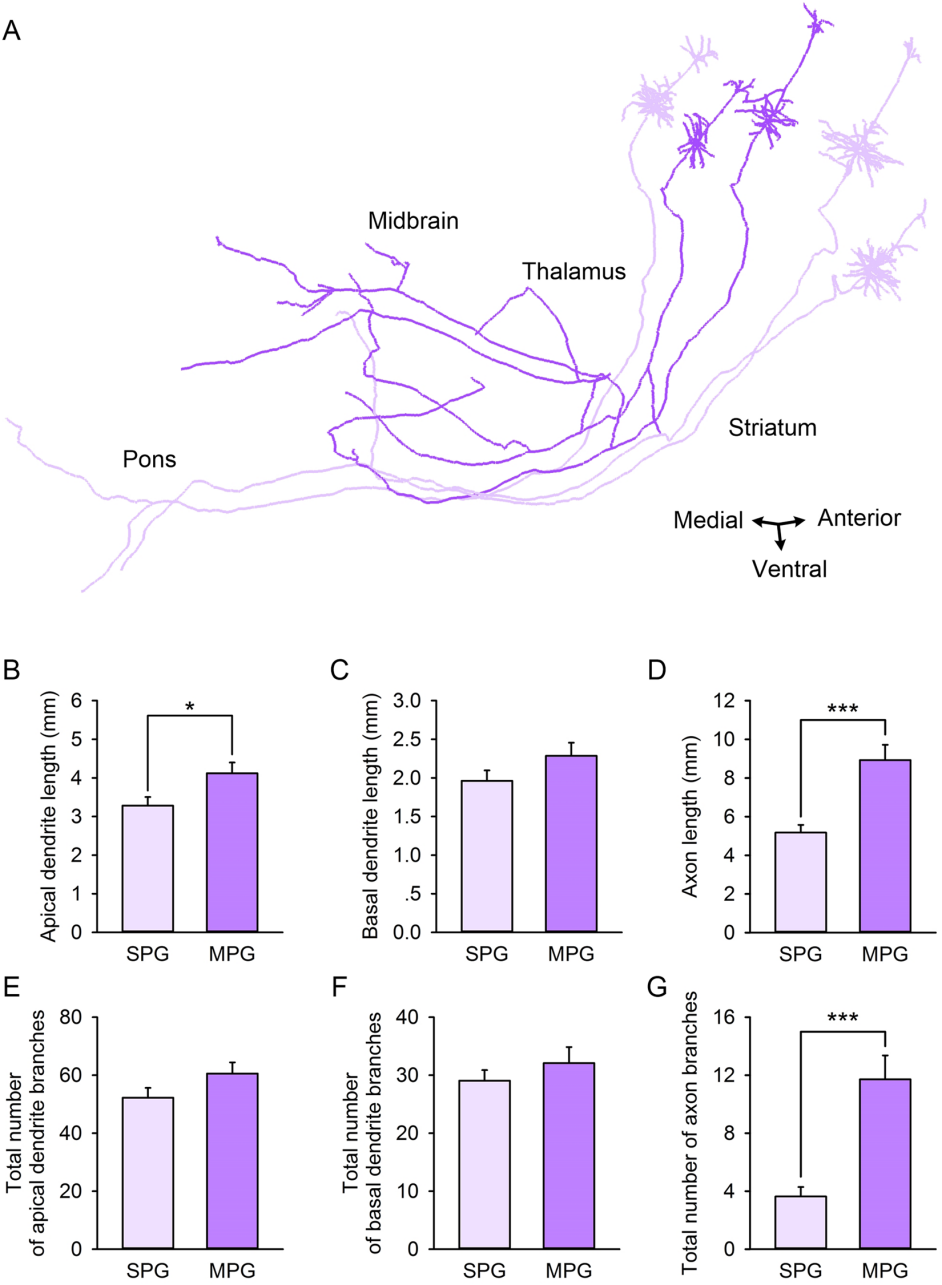
Comparative complexity of dendrites and axons between the single-region projection group (SPG) and the multi-region projection group (MPG). (**A**) Reconstruction samples of single-region projection neurons (n = 3, lavender) and multi-region projection neurons (n = 2, purple). Comparisons of apical dendrite length (**B**), basal dendrite length (**C**), axon length (**D**), total number of apical dendrite branches (**E**), total number of basal dendrite branches (**F**), and total number of axon branches (**G**) between the SPG and the MPG. SPG: n = 39; MPG: n = 28. *Represents *p*-*value* < 0.05, *** Represents *p*-*value* < 0.001.

Furthermore, we separated the L5 type I neurons into the single-region projection group (SPG) and the multi-region projection group (MPG). We measured and compared the dendrite complexities of the two groups (Fig. 5, Table 2) to examine whether a significant difference existed between them.

**Table 2:** Comparison of morphology measures between the SPG and the MPG.

	Apical dendrite lenth (μm)*	Basal dendrite length (μm)	Axon lengh (μm)***	Total number of apical dendrite branches	Total number of basal dendrite branches	Total number of axon branches***
SPG	3344.4 ± 244.4	2012.5 ± 158.6	5176.4 ± 400.2	53.5 ± 3.8	29.8 ± 2.3	3.6 ± 0.7
MPG	4131.7 ± 302.6	2339.9 ± 209.0	8924.0 ± 791.8	60.9 ± 4.2	33.1 ± 3.3	11.7 ± 1.7

SPG: n = 39; MPG: n = 28. *Represents *p*-*value* < 0.05, *** represents *p*-*value* < 0.001 (two-tailed).

Statistical results demonstrated that the average apical dendrite length of the MPG was greater than the SPG (Fig. 5B, *p*-value = 0.045, two-tailed), but branch number of basal and apical dendrites and average basal dendrite length did not show statistical significances between SPG and MPG (Fig. 5C–E). Morphological measures indicated that the average length of apical dendrites of the MPG was greater than that in the SPG. This result suggests that there is a certain degree of correlation between the number of projection directions and dendrite complexity.

**Figure 6:**
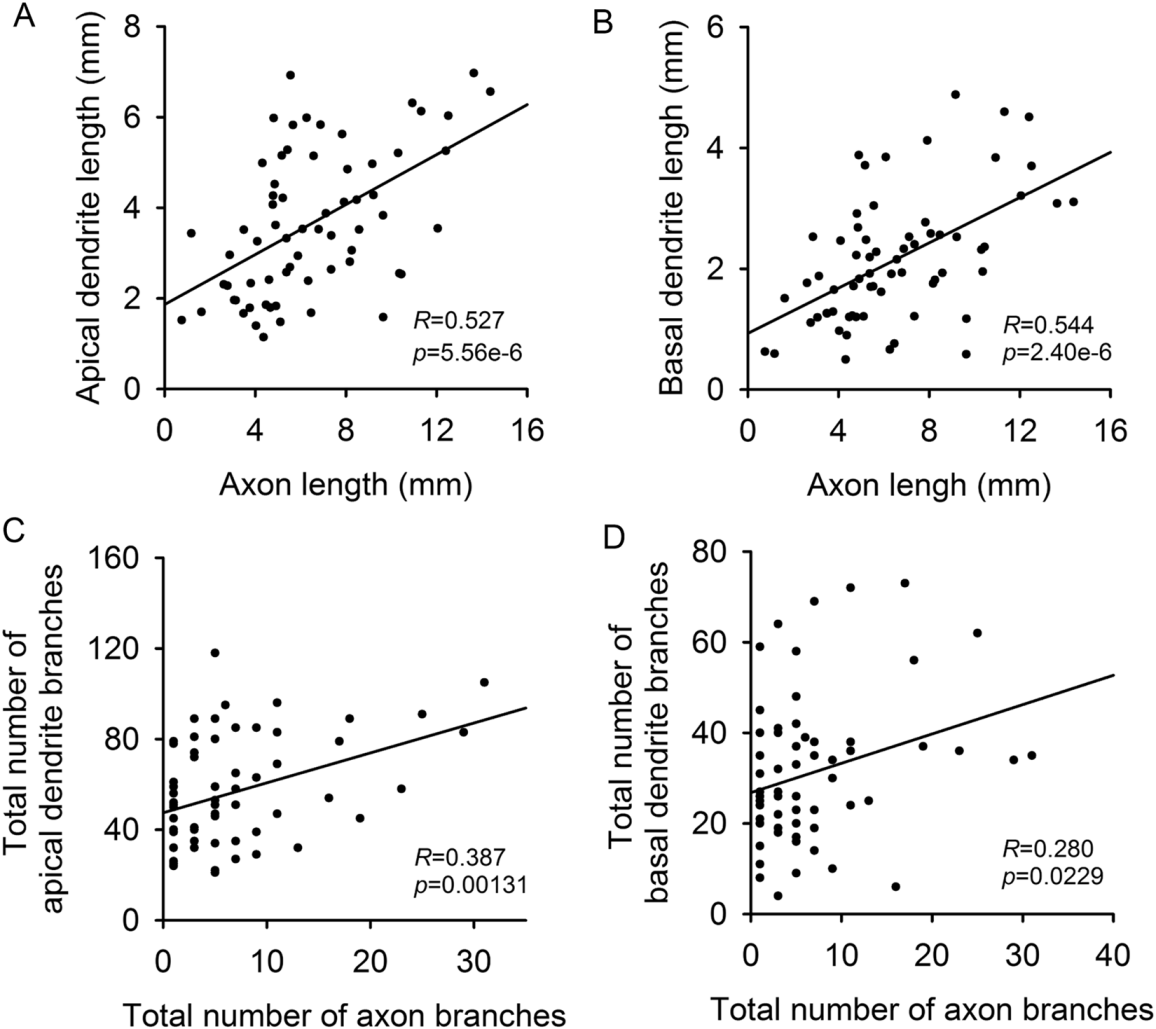
Linear correlations of the axonal and dendritic complexity parameters of L5 type I neurons, where *R* represents the Pearson correlation coefficient and *p* represents the *p*-*value* (two-tailed).

A subsequent linear correlation analysis directly demonstrated the above inference. The axon length was moderately correlated with apical and basal dendrite length in L5 type I neurons (Fig. 6A and B), but the total number of axon branches only had low correlations with those in dendrites (Fig. 6C and D).

## Discussion

We analyzed the morphology of corticofugal projection neurons of a Thy1-GFP M-line mouse in layer V and VI of the barrel field by applying the brain-wide positioning system. With high axial resolution and natural cytoarchitecture registration, we acquired the projection distributions of several types of pyramidal neurons and the projection directions of every individual neuron. Focusing on L5 type I neurons, we analyzed the different projection modes among them to reveal the similarities and variances of multidirectional projections in different regions. The small sample size of the other type of neurons restricted further relevant statistical analysis. This should be performed with a variant-effective labeling method other than the transgenic labeling of Thy1-GFP.

In the barrel field of the Thy1-GFP M-line mouse, we observed three morphological divergent types of pyramidal neurons, including layer V neurons that possess abundant and high arbors of apical dendrites and layer VI neurons that occupied the majority. The main dendritic morphology patterns of the vast majority of L5 type I neurons is consistent with “thick-tufted layer V pyramidal neurons”^24^. The collective distribution of the corticofugal projections of L5 type I neurons is consistent with the previous conclusion in the barrel field, such as the thalamus, superior colliculus, pontine nuclei, zona incerta and caudate putamen^3, 25, 26^. It should be noted that due to the rapidly increasing difficulty of manual tracing in fiber tracts, the axon distal terminals in the cst and cc most likely kept moving in the direction of the medulla (also likely to the contralateral regions of the cc)^27^. L5 type II neurons have a similarity to the cortico-cortical pyramidal neurons in both layer 5 and the upper layer 6 in terms of dendritic morphology^3, 28–30^. Since we have observed the fine apical dendrite tufts of the type I, the missing of apical dendritic tuft reaching layer 1 of the type II may not be from systematic error. The uncertainty of the subclass of L5 type II should be clarified with more detailed morphological analysis in the future. The projection distances of the corticofugal projections of layer VI neurons were always relatively short. Although a few neurons project to the midbrain and even to the pons, the thalamus remains the prominent projection region. The projection frequency of the striatum increased in layer VI neurons, which is also consistent with previous studies^3, 29^.

The multidirectional projection ratio of L5 type I neurons was much larger than layer VI neurons. The number of multidirectional projection neurons of the latter was small, so we focused on the multidirectional projection neurons in the L5 type I group. The results suggest that the thalamus and midbrain are the principal regions in the composition of multidirectional projections. For all of the multidirectional projection neurons, the ratio of “extra projections” for the thalamus and midbrain is greater than 20% in all main projection regions, which indicates the significant role of both of them in the rodent whisker sensorimotor system. They have bidirectional connections between the VPM and POM of the thalamus and barrel field^31, 32^. As a main input source region of the barrel field^33^, the thalamus is a key component in signal conduction and feedback in the rodent whisker sensorimotor system. The superior colliculus, which belongs to the midbrain, could participate in the control of the vibrissae movements^34^. Moreover, the frequency of simultaneous projections to the thalamus and midbrain is very high, which suggests the strong collaboration of the thalamus and the midbrain.

We found that the hypothalamus always appears as an “additional projection” (Table 1). In contrast to the key functional situation of the thalamus and the midbrain, the hypothalamus may play an important assistant role in the rodent whisker sensorimotor system. Most of the distal terminals that we observed in the hypothalamus were located in zona incerta, which provides a disinhibitory mechanism in the somatosensory activities of the whiskers^35^. The value of the hypothalamus-thalamus in the collaboration matrix is larger than other region-region groups (Fig. 4A), which confirms the inhibitory effects from neurons in the zona incerta to the thalamocortical neurons in the high-order nuclei of thalamus^36, 37^.

In addition, the properties of multidirectional projection L5 type I neurons correlate with the complexity of dendrites, that is, the average branch length of apical dendrites is longer in the MPG. This structural correlation may be involved in the developmental process of L5 type I neurons with various functional differentiations. If multidirectional projection neurons have more complicated functions, as the receiving sites of cortical or subcortical projections, then the dendrites should be more “dense” to adapt to the multifunctional output. These results could contribute to studies on the computational simulation of neocortical circuits.

In summary, this pilot study targeted the single-axonal level projection analysis of corticofugal neurons in the barrel field. It provides direct observations on projection modes and the morphological correlation between dendrites and axons, which are not accessible at the population level. This work demonstrates a paradigm on the morphological analysis of neurite-level connectivity in specific-labeled neural circuits. In the future, we will use BPS to interpret refined projectomes across the whole mouse brain.

## Methods

### Tissue preparation

Two Thy1-GFP M-line transgenic mice (Jackson Laboratory, Bar Harbor, ME, USA) were used in this study. The mice were anesthetized with a 1% solution of sodium pentobarbital and were intracardially perfused with 0.01 M PBS (Sigma-Aldrich Inc., St. Louis, MO, USA) followed by 4% paraformaldehyde (Sigma-Aldrich Inc., St. Louis, MO, USA) and 2.5% sucrose in 0.01 M PBS. The brains were excised and post-fixed in 4% paraformaldehyde at 4 °C for 24 h. After fixation, each intact brain was rinsed overnight at 4 °C in a 0.01 M PBS solution that contained 2.5% sucrose and was subsequently dehydrated via immersion in a graded series of ethanol mixtures (1 h each at 4 °C). To increase the signal-noise ratio, we improved the previously used resin-embedding approach: following dehydration, each intact brain was impregnated with glycol methacrylate (GMA, Ted Pella Inc., Redding, CA, USA) using sequential 2-h immersions in 70%, 85%, 100% GMA and 100% GMA overnight at 4 °C. Here, a GMA solution (including 0.2% Sudan Black B, i.e., SBB) was prepared from 95% ethanol and 100% GMA (wt/wt). Subsequently, the samples were impregnated in a prepolymerization solution of GMA (including 0.2% SBB) for 3 days at 4 °C and were embedded in a vacuum oven. All of the animal experiments followed procedures that had been approved by the Institutional Animal Ethics Committee of Huazhong University of Science and Technology. And, animal care and use was done in accordance with the guidelines of the Administration Committee of Affairs Concerning Experimental Animals in Hubei Province of China. More detailed histological procedures have been described previously^38^.

### Whole-brain imaging with real-time propidium iodide (PI) staining

The mice brains were sectioned and imaged automatically using BPS^21^. Before imaging, the resin-embedded whole-brain sample was immobilized in the anterior-posterior direction in a water bath on a 3D translation stage. The water bath was filled with the PI-Na_2_CO_3_ solution, in which the sample was immersed. Sectioning was achieved using a fixed diamond knife and a 3D translation stage in the wide-field large-volume tomography. The x-axis of the translation stage was the sectioning direction, the single sectioning thickness was set to 4 μm, and the sectioning width was 2 mm. When the superficial layer of the sample block was removed, the new surface contacted the PI-Na_2_CO_3_ solution, which quickly penetrated the sample surface (approximately 10 μm depth). The PI molecules combined with the nucleic acids inside the cell body, revealing the soma and a portion of the dendrites and axon hillock. In contrast, Na_2_CO_3_ could enhance the GFP signal. Real-time counterstaining and the fluorescent protein signal enhancement were achieved simultaneously during the sectioning and imaging steps. The imaging was performed using a 20× water immersion objective on a fast structured illumination microscopy (1.0 NA, XLUMPLFLN 20XW, Olympus, Shinjuku, Tokyo, Japan). The imaging plane was set below the surface of the sample block. The GFP and PI molecules were excited simultaneously, and the emitted fluorescence signals were separated by a dichroic mirror and detected by two cameras. Three phase-shifted raw images were required to obtain an optical section image for each imaging channel. Axial scanning was then executed using the piezoelectric translational stage, which acquired two sectioning images at depths of 2 μm and 4 μm. Following axial scanning, the sample was moved to the next mosaic field of view (FOV), with a 10 μm overlap between adjacent FOVs. The mosaic imaging process was repeated until the entire coronal section was acquired.

### Image preprocessing

The image preprocessing intends to obtain a standard dataset for both the GFP and PI channels, as follows. First, the tiles of the same section were stitched to obtain a mosaic section based on the accurate spatial orientation and neighboring overlap (approximately 10 pixels). The anchor points of the tiles were spaced equally in two orthogonal directions. Second, transverse illumination correction was performed separately in each section. Third, axial illumination correction was performed based on the average intensity of each section. The two illumination correction steps were based on our previously developed algorithm^39^. Image preprocessing was implemented in C++ and parallel optimized using the Intel MPI Library and then executed on a computing server (72 cores, 2 GHz/core) within 6 h for each mouse brain dataset at a voxel resolution of 0.32 × 0.32 × 2 μm^3^.

### Reconstruction and statistics

We determined the radial bounding box and the axial slice section where the barrel field was located according to the Allen Mouse Brain Reference Atlas in the PI channel image series from every Thy1-GFP M-line dataset (Fig. 1A). Consistent with previous work^21^, we chose the pyramidal neurons at layer V or VI for tracing and reconstruction (Fig. 1B). Brain-wide tracing for neurites was applied in the filament editor module of Amira (FEI, Mérignac Cedex, France) in 3D by a human-machine interaction. If no reconstructed axon collateral of one pyramidal neuron reached the corpus callosum, then the neuron was eliminated from the candidates of the subsequent corticofugal projection analysis. All morphology parameters were measured using Neurolucida Explorer (MBF Bioscience, Williston, VT, USA). We performed Student’s *t*-test to confirm the classification of L5 neurons and the complexity correlation of the projection-dendrite using SPSS software (v 22, IBM, New York, USA). The confidence level was set to 0.05 (alpha). All error bars and the values that followed with “±” were defined as the s.e.m.

## Electronic supplementary material

Projection information on L5 type II and L6 neurons - SUPPLEMENTARY INFORMATION
