# Supplementary material for: Single-axon level morphological analysis of corticofugal projection neurons in mouse barrel field
Source: Sci Rep. 2017 Jun 6;7:2846. doi: 10.1038/s41598-017-03000-8 (PMC5460143; doi:10.1038/s41598-017-03000-8)
Supplement: Supplementary file 1 — Projection information on L5 type II and L6 neurons - SUPPLEMENTARY INFORMATION [file 41598_2017_3000_MOESM1_ESM.doc]

**Single-axon level morphological analysis of corticofugal projection neurons in mouse barrel field**

Congdi Guo^1,2^, Jie Peng^1,2^, Yalun Zhang^1,2^, Anan Li^1,2^, Yuxin Li^1,2^, Jing Yuan^1,2^, Xiaofeng Xu^1,2^, Miao Ren^1,2^, Hui Gong^1,2^, Shangbin Chen^1,2^

*^1^Britton Chance Center for Biomedical Photonics, Wuhan National Laboratory for Optoelectronics-Huazhong University of Science and Technology, Wuhan 430074, China.*

*^2^Key Laboratory for Biomedical Photonics of Ministry of Education, Department of Biomedical Engineering, Huazhong University of Science and Technology, Wuhan 430074, China.*

*Correspondence should be addressed to S.C. (sbchen@mail.hust.edu.cn).*

| **No.** | **Projection region** | **Projection mode** |
| --- | --- | --- |
| **1** | CP, TH | ST |
| **2** | CP | S |
| **3** | int | C |
| **4** | CP | S |
| **5** | int | C |
| **6** | cc | - |
| **7** | int | C |
| **8** | int | C |
| **9** | TH | T |

Supplementary Table S1. Projection region and projection mode of all L5 type II neurons.

| **No.** | **Projection region** | **Projection mode** |
| --- | --- | --- |
| **1** | TH | T |
| **2** | TH | T |
| **3** | CP | S |
| **4** | TH | T |
| **5** | TH | T |
| **6** | cc | - |
| **7** | P | P |
| **8** | TH | T |
| **9** | TH | T |
| **10** | cpd | C |
| **11** | TH | T |
| **12** | CP | S |
| **13** | TH | T |
| **14** | cc | - |
| **15** | TH | T |
| **16** | TH | T |
| **17** | MB | M |
| **18** | cc | - |
| **19** | TH | T |
| **20** | TH, P | TP |
| **21** | cc | - |
| **22** | TH | T |
| **23** | TH | T |
| **24** | cpd, TH | CT |
| **25** | TH | T |
| **26** | CP | S |
| **27** | int, cc | C |
| **28** | int | C |
| **29** | CP | S |
| **30** | TH | T |
| **31** | cpd | C |

Supplementary Table S2. Projection region and projection mode of all L6 neurons.

**Abbreviations:**

cc - corpus callosum;

CP – Caudoputamen;

cpd - cerebal peduncle;

int - internal capsule;

MB – Midbrain;

TH –Thalamus;

P – Pons;
